# Intercropping Walnut and Tea: Effects on Soil Nutrients, Enzyme Activity, and Microbial Communities

**DOI:** 10.3389/fmicb.2022.852342

**Published:** 2022-03-18

**Authors:** Yong-Chao Bai, Bao-Xin Li, Chun-Yong Xu, Mubashar Raza, Qi Wang, Qi-Zhu Wang, Ya-Nan Fu, Jian-Yang Hu, Abdessamad Imoulan, Muzammil Hussain, Yong-Jie Xu

**Affiliations:** ^1^State Key Laboratory of Tree Genetics and Breeding, Key Laboratory of Tree Breeding and Cultivation of the State Forestry and Grassland Administration, Research Institute of Forestry, Chinese Academy of Forestry, Beijing, China; ^2^Hubei Academy of Forestry, Wuhan, China; ^3^State Key Laboratory of Mycology, Institute of Microbiology, Chinese Academy of Sciences, Beijing, China; ^4^Center for Walnut Technology of Baokang County, Xiangyang, China; ^5^State Key Laboratory of the Discovery and Development of Novel Pesticides, Shenyang Sinochem Agrochemicals R&D Co., Ltd., Shenyang, China; ^6^Department of Biology, Faculty of Science and Technics of Errachidia, Mouly Ismail University, Meknes, Morocco

**Keywords:** intercropping, soil nutrients, bacterial community, fungal community, microbial diversity, beneficial microbiota

## Abstract

The practice of intercropping, which involves growing more than one crop simultaneously during the same growing season, is becoming more important for increasing soil quality, land-use efficiency, and subsequently crop productivity. The present study examined changes in soil physicochemical properties, enzymatic activity, and microbial community composition when walnut (*Juglans* spp.) was intercropped with tea (*Camellia sinensis* L.) plants in a forest and compared with a walnut and tea monocropping system. The results showed that walnut–tea intercropping improved the soil nutrient profile and enzymatic activity. The soil available nitrogen (AN), available phosphorus (AP), available potassium (AK), organic matter (OM) content, and sucrase activity were significantly boosted in intercropped walnut and tea than in monocropping forests. The interaction between crops further increased bacterial and fungal diversity when compared to monoculture tea forests. Proteobacteria, Bacteroidetes, Firmicutes, Chlamydiae, Rozellomycota, and Zoopagomycota were found in greater abundance in an intercropping pattern than in monoculture walnut and tea forest plantations. The walnut–tea intercropping system also markedly impacted the abundance of several bacterial and fungal operational taxonomic units (OTUs), which were previously shown to support nutrient cycling, prevent diseases, and ameliorate abiotic stress. The results of this study suggest that intercropping walnut with tea increased host fitness and growth by positively influencing soil microbial populations.

## Introduction

Walnut (*Juglans* spp.) is a perennial deciduous angiosperm that has gained increasing attention in recent years due to its economic importance. It is primarily grown for nut and wood products but is also an attractive ornamental tree in parks (Mortier et al., 2020). Recently, many farmlands have been transformed into walnut orchards in an attempt to increase walnut production and profitability (Bai et al., 2020). The farmers found that growing maize, wheat, or vegetables beneath the walnut trees increased output and revenue (Zhang W. et al., 2014; Pardon et al., 2019). Walnut trees can also positively affect crops physiology (Zhang W. et al., 2014). It has been previously shown that a combination of forest trees and crops improves land-use efficiency, reduces weed pressure, optimizes soil temperature, and conserves soil moisture (Tsonkova et al., 2012; Zhang D. S. et al., 2014; Gebru, 2015; Torralba et al., 2016; Panozzo et al., 2019). Forest trees regulate the climate underneath them by providing shelter and limiting soil evaporation (Lasco et al., 2014; Pardon et al., 2017). It may result in changes in soil microclimatic factors, such as humidity and temperature, when compared to an open field (Lott et al., 2009). In China, tea (*Camellia sinensis* L.) is also widely intercropped with walnut trees in the southwest region and the Daba Mountains (Wang et al., 2014; Tang et al., 2021). Although walnut trees compete with tea for soil resources in intercropping systems, they can also improve the growing conditions of tea trees, especially by providing moderate shade and increasing humidity in spring (Kulasegaram and Kathiravetpillai, 1976). Several studies have examined intercropping between trees and annual crops (Zhang W. et al., 2014; Arenas-Corraliza et al., 2018; Pardon et al., 2019; Temani et al., 2021); however, very little research has been conducted on intercropping between perennial non-wood forest trees (walnut) and crops. Additionally, the effects of tree–crop interactions on soil microbial diversity and community composition are largely unknown.

Soil microbiota are an important component of both natural and managed ecosystems (Fierer, 2017). Crop management practices that shape soil microbial communities under field conditions have greatly improved our understanding of how management factors affect crop production, biogeochemical cycling, and disease progression (Berthrong et al., 2013; Hussain et al., 2018; Raza et al., 2019; Tao et al., 2020). Therefore, microbial communities in relation to management strategies have become an imperative aspect of research in sustainable agriculture (Lacombe et al., 2009; Bainard et al., 2012b; Zhang M. M. et al., 2019). Intercropping, growing two crops simultaneously in proximity, not only affects crop yield but may also change the structure and functions of the soil microbiota (Duchene et al., 2017; Yu et al., 2019). In intercropping systems, soil nutrients, enzymes, and microbes interact to enhance the micro-ecological conditions of the soil by increasing the microbial diversity and enzyme activity (Zhou et al., 2019). Microorganisms influence soil nutrient turnover by decomposing soil organic matter (OM), which in turn influences soil enzyme properties and enzyme secretion (Peng et al., 2015). Soil enzymes (e.g., catalase, urease, and invertase) catalyze biochemical reactions during the decomposition of microorganisms and plants debris, which provide the soil with nutrients that plants need to survive (Veres et al., 2015). Therefore, the soil enzyme activity is closely linked to microbiological characteristics for improving soil-plant health (Cleveland and Liptzin, 2007). In previous studies, intercropping peanuts with maize changed the abundance of nitrogen-fixing microbes in the rhizosphere (Chen et al., 2018). Cassava–peanut intercropping enriched for Actinomycetes in the rhizosphere of peanut that boosted soil available nutrients absorption, increasing the peanut yield (Chen et al., 2020). It is therefore necessary to conduct further research to understand how tree–crop intercropping affects soil nutrient content, enzyme activities, and microbial community diversity and structure that would be crucial for evaluating the nutrient status and energy outputs of soil microbes in forest ecosystems.

Since various intercropping patterns differentially affect soil physicochemical properties and microbial characteristics, this study examined the changes in the bacterial and fungal communities as a result of the walnut–tea intercropping forest system. We hypothesized that intercropping would greatly affect soil physicochemical properties, increase enzyme activities and microbial diversity, and change the microbial community structure. Thus, the objectives of this study were to (1) explore the effects of walnut–tea intercropping on soil physicochemical properties and soil enzyme activity; (2) compare the response of bacterial and fungal diversity and community composition to intercropping with monoculture plantations of walnut and tea; and (3) determine the relationship between soil microbial communities and soil physicochemical properties and soil enzyme activity.

## Materials and Methods

### Site Description and Soil Sampling

**FIGURE 1:**
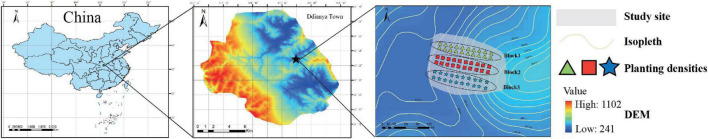
A map of sampling locations in the experimental areas of the Daba Mountains. A diagram showing the collection of soil samples from monoculture tea (T) and walnut (W) forests and intercropping forests (W&T) in Dianya town. Green triangles, red squares, and blue stars represent monoculture tea forests (T), monoculture walnut forests (W), and intercropping forests (W&T) in study sites, respectively.

Walnut and tea forest sites are located at Dianya, Baokang County, Hubei Province, China (31° 25′28″N, 111° 21′58″E). In this region, the average temperature is 17°C, the mean annual relative humidity is 71.2%, the average annual precipitation is 1,071 mm, and the frost-free season is 240 days. The forest soils are yellow-brown earth with sandy loam texture, the slope is 10°, and the elevation is 538 m (Figure 1).

We selected three types of forest that include monoculture tea forest (T), monoculture walnut forest (W), and walnut–tea intercropping forests (W&T) to investigate how they influence soil properties, enzyme activity, and microbial community composition. In the spring of 1992, a tea forest was established by sowing local tea seeds at a row spacing of 1.3∼1.5 m, cluster spacing of 25∼33 cm, and 2∼3 plants per cluster. In the spring of 2009, a walnut forest was established with 1-year-old grafted seedlings of the variety “Qingxiang” at a plant row spacing of 4 m × 8 m. Similarly, an intercropping forest was also established in the spring of 2009 by planting the 1-year-old grafted seeds of “Qingxiang” in the 17-year-old pure tea forest based on the plant row spacing of 4 m × 8 m. Each forest soil received fertilizer twice a year. For the first time, organic fertilizer (compost of pig manure; 11,250 kg⋅hm^–2^, broadcast fertilization) was broadcast over the plant row in each forest and then covered with 5∼8-cm-thick corn stalks. The second time, in May, each forest received 375 kg⋅hm^–2^ of urea.

We collected soil samples from each forest type using augers (5 cm in diameter) at a depth of 20∼30 cm following the S-sampling method. Ten cores of soil per forest were mixed thoroughly in order to represent one replicate, and three replicates were collected from each forest. The soil samples from each forest were sieved using a 2-mm mesh and transported to the laboratory for further study.

### Soil Physicochemical Analyses

The chemical properties of soil samples were determined using air-dried samples. Soil pH was determined with a pH meter (FE-20, Swiss Mettler, Zürich, Switzerland) based on a soil-to-water ratio of 1:2.5. Available nitrogen (AN) content of the soil was measured using the DigiPREP TKN System (KJELTEC 8400, Foss, Denmark). Available phosphorus (AP) was measured using a UV-visible spectrophotometer (UH5300, North Points Ruili). Available potassium (AK) was quantified from soil using an inductively coupled plasma (ICP) spectrometer (Spectro Analytical Instruments, Spectro Arcos ICP, Kleve, Germany). Soil OM content was quantified using the K_2_Cr_2_O_7_–H_2_SO_4_ oxidation method.

### Soil Enzyme Activity Assay

We quantified the soil enzymes urease (UE), alkaline phosphatase (ALP), peroxidase (POD), and sucrase (SC) involved in nitrogen, carbon, and phosphorus degradation. The soil urease activity was measured using urea as the substrate as described by Bao (2000). A soil ALP assay was performed according to the protocol of Dick et al. (2000). The soil peroxidase was measured in a 96-well microplate using the spectrophotometric method with L-3,4-dihydroxyphenylalanine (L-DOPA) as substrate (Bach et al., 2013). The soil sucrase activity was determined by measuring the glucose released from a sucrose solution (8%) after incubation at 37°C for 24 h (Chen et al., 2010).

### DNA Extraction and Illumina MiSeq Sequencing

Total soil DNA was extracted from 0.5 g of soil from each sample using a Power Soil DNA Kit (MOBIO Inc., Carlsbad, CA, United States), according to the manufacturer’s instructions. PCR was performed to amplify the V3–V4 region of the bacterial 16S rRNA gene using primer pair 338F 5′-ACTCCTACGGGAGGCAGCA-3′ and 806 R 5′-GGACTACHVGGGTWTCTAAT-3′. For the fungal community, the ITS1 region of ITS gene was amplified using primer pair ITS5 5′-GGAAGTAAAAGTCGTAACAAGG-3′ and ITS2 5′-GCTGCGTTCTTCATCGATGC-3′. PCR generated DNA amplicons were purified using the AxyPrepDNAGel Extraction Kit (AXYGEN, Union City, CA, United States) and pooled in equimolar concentrations. Finally, paired-end sequencing of the bacteria and fungi was performed on an Illumina MiSeq sequencer at Majorbio Technologies Co., Ltd. (Shanghai, China). The raw sequence data have been deposited in the National Genomics Data Center under BioProjectID PRJCA008251.

### Bioinformatics and Statistical Analyses

The paired-end reads were initially trimmed using Mothur (V1.30.2) to remove sequences with a quality score below 20. The 16S rRNA and ITS1 sequences were quality-trimmed using Trimmomatic v0.36 and assigned to samples based on barcodes using Quantitative Insights into Microbial Ecology (QIIME, V1.9.1). A number of *de novo* and reference-based chimeras were checked, and those identified as chimeras were eliminated. Bacterial and fungal sequences were grouped into operational taxonomic units (OTUs) based on 97% sequence similarity using UPARSE-pipeline (V 7.0.1090), and the most abundant sequences from each OTU were selected as representative sequences. The taxonomic configuration of bacterial and fungal OTUs was done using the SILVA (V132) and UNITE (V8.0) databases, respectively. Alpha diversity metrics were computed with R package “vegan,” and the results were visualized in boxplots using the R package “ggplot2.” In order to examine similarities and differences between bacterial and fungal communities, a beta-diversity analysis based on the Bray–Curtis dissimilarity matrix was calculated using the function “vegdist” in the R package “vegan.” Based on the classified OTU reads, relative abundance (RA) (‰) of the abundant phyla and genera was determined and plotted using the R package “ggplot2.” An ANOVA was performed to compare soil physicochemical properties, diversity metrics, and taxonomic composition of bacterial and fungal communities. The least significant difference (LSD) test was used to distinguish differences between groups and was considered significant when *p* < 0.05. We identified enriched bacterial and fungal OTUs in tea forest, walnut forest, and walnut–tea intercropping forest and visualized them in ternary plots using a script developed by Bulgarelli et al. (2015), which employed linear statistics on the RA values (log2, >1‰ threshold) using the R package “limma.” Differentially abundant OTUs between groups were calculated using a moderated *t*-test, and the obtained *p*-values were adjusted using the Benjamini–Hochberg correction method. Enriched OTUs with taxonomic information were presented in heatmaps using the heatmap.2 function in the R package “gplots.”

## Results

### Impact of Forest Types on Soil Physicochemical Properties and Enzymatic Activity

**FIGURE 2:**
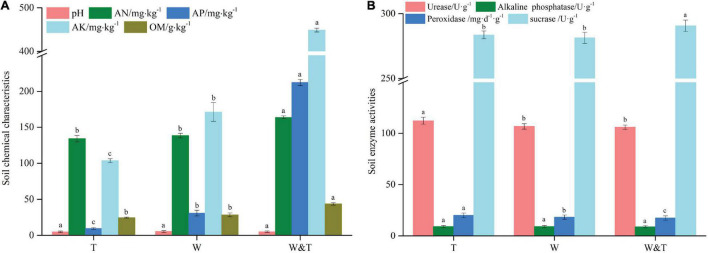
Chemical properties **(A)** and enzyme activities **(B)** of the pure forest and intercropping forest. A different lowercase letter on each bar indicates a least significant difference (LSD; *p* < 0.05) between monoculture tea forest (T), monoculture walnut forest (W), and walnut–tea intercropping forest (W&T). AN, available nitrogen; AP, available phosphorus; AK, available potassium; OM, organic matter.

In the three forest types (Figure 1), the walnut–tea intercropping forest (W&T) had significantly higher levels of AN (164.1 mg⋅kg^–1^), AP (212.3 mg⋅kg^–1^), AK (449.1 mg⋅kg^–1^), and OM (43.6 g⋅kg^–1^) than the monoculture walnut and tea forest (LSD, *p* < 0.05, Figure 2A). These nutrient contents were higher than those of monoculture tea forest by 22.3% (AN), 2,131.9% (AP), 333.1% (AK), and 77.2% (OM) and monoculture walnut forest by 18.5% (AN), 587.9% (AP), 162.1% (AK), and 53.7% (OM). Surprisingly, the intercropped forest had the highest level of soil AK, followed by AP and AN. Among monoculture forests, this nutrient enrichment pattern was different from intercropped forests. Walnut forest had higher levels of AK and AN followed by AP, while tea forest had higher levels of AN followed by AK and AP. Although forest types did not affect soil pH, the maximum pH value was found in walnut forest, followed by walnut-tree intercropped (pH = 5.0), and monoculture tea forest (pH = 4.8) (LSD, *p* > 0.05, Figure 2A).

We then examined the soil enzymatic activities and found that the intercropped forest had a high sucrase activity (SC) in comparison with the walnut and tea forests. Peroxidase activity (POD) was significantly higher in tea forest than in walnut forest and in the walnut–tea intercropping forest, while ALP activity was not statistically different between forest types (Figure 2B). The soil urease activity (UE) was the highest in the tea forest and not significantly different between the intercropped and walnut forests.

### Intercropping Affects Bacterial Diversity and Community Structure

**FIGURE 3:**
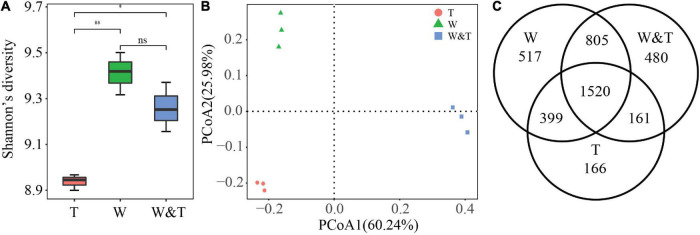
Bacterial community α-diversity and β-diversity. **(A)** Shannon’s diversity for bacterial communities in monoculture tea forest (T), monoculture walnut forest (W), and walnut–tea intercropping forest (W&T). A box indicates the interquartile range; a black line indicates the median value. A lowercase letter on each box represents a least significant difference (LSD; *p* < 0.05) between the T, W, and T&W. Asterisks indicates significant differences (*p < 0.05, ^**^p < 0.01). **(B)** Principal coordinate analysis (PCoA) plots based on the Bray–Curtis distance demonstrating the separation between soil bacterial communities of three forest types. **(C)** The Venn diagram shows the numbers of bacterial operational taxonomic units (OTUs) that are shared or unshared by T, W, and W&T.

A total of 454,258 high-quality reads targeting the 16S rRNA V3–V4 region of bacteria were obtained by sequencing the amplicons on an Illumina MiSeq sequencer. These reads were clustered into 4,048 OTUs. First, we assessed the within-sample diversity (α-diversity) of bacterial communities from monoculture walnut (W), monoculture tea (T), and walnut–tea intercropping forest (W&T). Shannon’s diversity values for bacterial communities decreased in the order W > W&T > T. Compared to monoculture tea forests, intercropped and monoculture walnut forests had significantly higher Shannon diversity values (LSD, *p* < 0.05, Figure 3A). However, we did not find a significant difference between W and W&T (LSD, *p* > 0.05). A principal coordinate analysis (PCoA) based on the Bray–Curtis distance was performed to analyze the differences among bacterial communities in three forest types. A clear separation could be observed between monoculture tea forest (T), monoculture walnut forest (W), and intercropped forest (W&T). According to PCoA results, the first two axes explain 60.24 and 25.98% of the total variation in the bacterial community (Figure 3B). A Venn diagram further confirmed that the variation between bacteria communities of three forest types might be due to the change in the composition of many shared as well as unique OTUs (Figure 3C).

**FIGURE 4:**
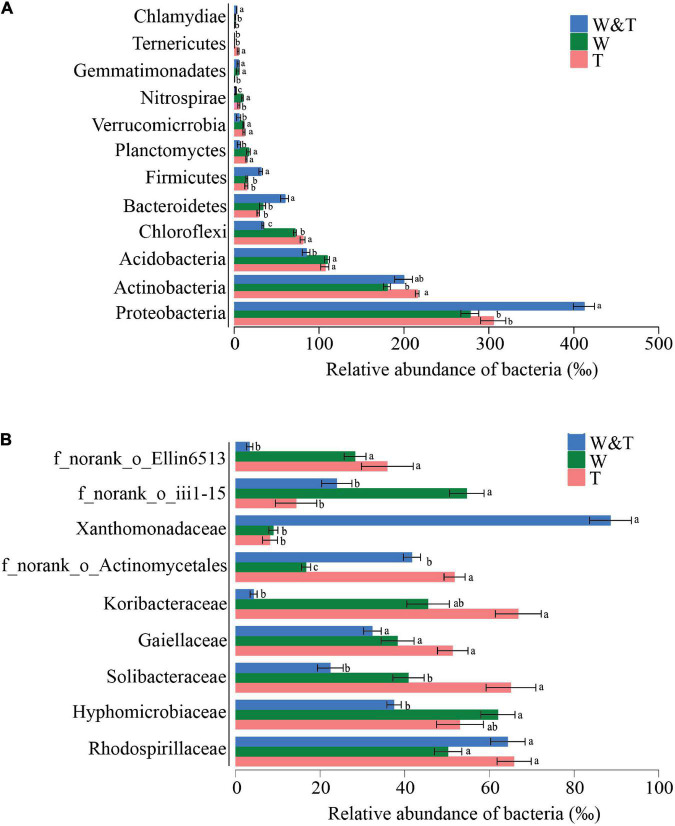
Average relative abundance (RA) of the most dominant bacterial phyla and families in the monoculture tea forest (T), monoculture walnut forest (W), and walnut–tea intercropping forest (W&T). **(A)** RA of bacterial communities at the phylum level. **(B)** RA of bacterial communities at the family level. Only operational taxonomic units (OTUs) with RA > 1% in at least one sample were included in the analysis. Different lowercase letters on each bar indicate the least significant differences (LSDs; *p* < 0.05) among T, W, and W&T treatments.

The phyla Proteobacteria, Actinobacteria, Acidobacteria, Chloroflexi, and Bacteroidetes dominated the soil bacterial communities in three forest types, accounting for more than 65% of the bacterial reads. The RA of Proteobacteria, Bacteroidetes, Firmicutes, and Chlamydiae was significantly higher in the intercropped forest than in monoculture tea or walnut forests (LSD, *p* < 0.05; Figure 4A). In contrast, the RA of Acidobacteria, Chloroflexi, Planctomycetes, and Tenericutes were more abundant in tea forests compared with walnut–tea intercropping forests (LSD, *p* < 0.05). There were several phyla unique to the walnut forest, such as Gemmatimonadetes and Chlamydiae, which were only more abundant in W&T and W. At the family level, patterns of taxonomic distribution and abundance differences became more evident (Figure 4B). Xanthomonadaceae was specifically dominant and significantly abundant in the walnut–tea intercropping compared to the monoculture forests. Monoculture tea and walnut forests contained high RAs of Solibacteraceae and some unclassified families.

### Fungal Community Diversity and Structure in Three Forest Types

**FIGURE 5:**
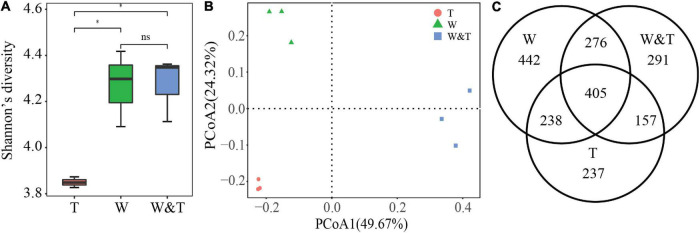
Fungal community α-diversity and β-diversity. **(A)** Shannon’s diversity for fungal communities in monoculture tea forest (T), monoculture walnut forest (W), and intercropping forest (W&T). A box indicates the interquartile range; a black line indicates the median value. A lowercase letter on each box represents a least significant difference (LSD, *p* < 0.05) between the T, W, and W&T. Asterisks indicates significant differences (*p < 0.05). **(B)** Principal coordinate analysis (PCoA) plots based on the Bray–Curtis distance demonstrating the separation between soil fungal communities of three forest types. **(C)** The Venn diagram shows the numbers of fungal operational taxonomic units (OTUs) that are shared or unshared by T, W, and W&T.

We obtained a total of 636,610 reads targeting the fungal ITS1 region, which were classified into 2,046 OTUs. Similar to the results for the bacterial community, the α-diversity results showed that Shannon’s diversity values for the fungal community were also significantly higher in the intercropped and monoculture walnut forests than in the tea forest (Figure 5A, LSD, *p* < 0.05). In addition, there was no significant difference in Shannon’s diversity values between W and W&T for the fungal community (LSD, *p* > 0.05). A PCoA based on Bray–Curtis distance revealed that the W&T, W, and T samples were clearly separated from each other, with the first two axes explaining 49.67 and 24.32% of the total variation (Figure 5B). Venn diagrams showed that the W&T, W, and T samples contained many unique OTUs and also shared 405 OTUs (Figure 5C). Hence, the variation in fungal community structure across W&T, W, and T might be linked to changes in the composition of the core and unique OTUs.

**FIGURE 6:**
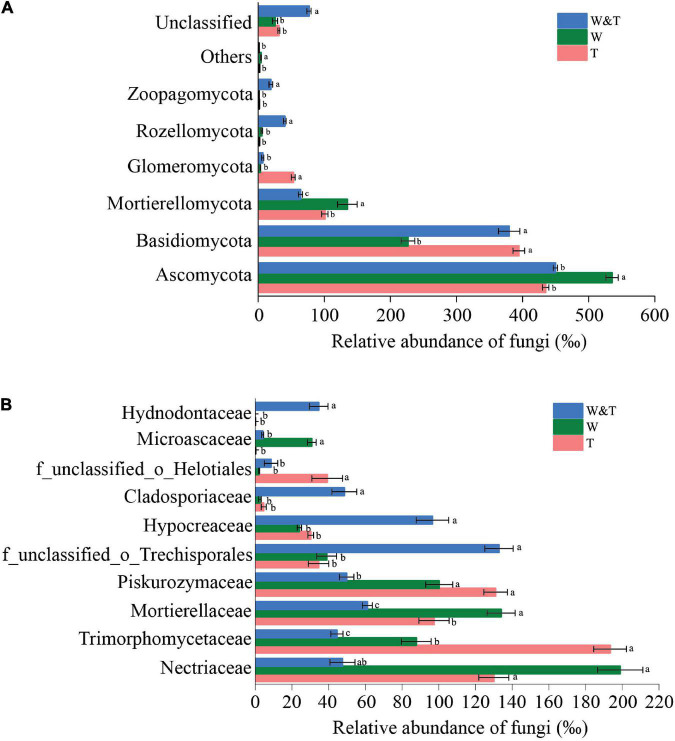
Average relative abundance (RA) of the most dominant fungal phyla and families in the monoculture tea forest (T), monoculture walnut forest (W), and walnut–tea intercropping forest (W&T). **(A)** RA of fungal communities at the phylum level. **(B)** RA of fungal communities at the family level. Only operational taxonomic units (OTUs) with RA > 1% in at least one sample were included in the analysis. Different lowercase letters on each bar indicate the least significant differences (LSDs; *p* < 0.05) among T, W, and W&T treatments.

The soil fungal community in three forest types was dominated by Ascomycota, Basidiomycota, Mortierellomycota, and Glomeromycota (Figure 6A). Ascomycota and Mortierellomycota had significantly higher RAs in monoculture walnut forests than in monoculture tea forests (LSD, *p* < 0.05). In contrast, the RA of Basidiomycota and Glomeromycota was significantly higher in T than in W (LSD, *p* < 0.05). Surprisingly, Rozellomycota and Zoopagomycota were significantly more abundant in W&T than in T and W. The families Hypocreaceae, Cladosporiaceae, and Hydnodontaceae were more abundant in W&T than W and T forests (Figure 6B), whereas the RA of Mortierellaceae and Microascaceae was higher in walnut forest soils than in W&T and T. Overall, the enriched bacterial phyla and families were different in the three forest types, indicating that intercropping had a differential impact on the soil fungal communities.

### Specific Soil Bacteria and Fungi Enriched in Walnut–Tea Intercropping Forest

**FIGURE 7:**
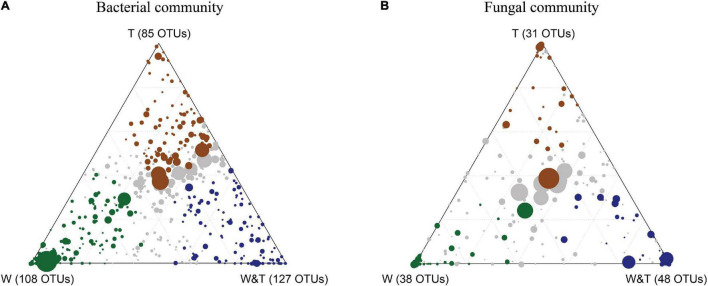
Ternary plot showing bacterial operational taxonomic units (OTUs) **(A)** and fungal OTUs **(B)** significantly enriched in monoculture tea forest (T, brown circles), monoculture walnut forest (W, green circles), and walnut–tea intercropping forest (W&T, blue circles). Each circle represents one OTU. The size of each circle represents its RA. The position of each circle is determined by the contribution of the indicated group to total RA. Only taxa with RA > 1‰ in at least one sample were included in the analysis.

The OTU enrichment analysis was performed to decipher which bacterial and fungal taxa in soil are responsive to specific forest types (Figures 7A,B). In total, 85 bacterial OTUs were identified to be significantly enriched in the monoculture tea forest, 108 bacterial OTUs in the monoculture walnut forest soils, and 127 bacterial OTUs in the walnut–tea intercropping forest type. These OTUs belong to diverse genera and phyla, as shown in the heatmaps in Supplementary Figures 1A–C. A number of OTUs were highly abundant in the walnut–tea intercropping forest, including *Chujaibacter*, *Acidothermus*, *Acidipila*, *Micropepsaceae*, *Bryobacter*, *Burkholderia*, *Pseudomonas*, and *Pseudolabrys*. On the other hand, there were only a few dominant OTUs more enriched in tea forests than walnut–tea intercropping forests, such as *Bradyrhizobium*, *Acidothermus*, and *Candidatus solibacter*. The functional role of dominantly enriched bacterial OTUs in W&T, W, and T is described in Supplementary Table 1.

In the fungal community, a total of 31 OTUs were enriched in T, 38 OTUs in W, and 48 OTUs in the W&T forest (Figure 7B). In walnut–tea intercropping, *Trichoderma*, *Trechispora*, *Talaromyces*, and *Penicillium* were predominant and enriched compared to other forest types (Supplementary Figure 2A). In contrast, the walnut forest had enriched OTUs belonging to *Neocosmospora*, *Lycoperdon*, *Solicoccozyma*, *Fusicolla*, *Lophotrichus*, *Pseudaleuria*, *Mortierella*, and *Phoma* (Supplementary Figure 2B). Tea forest soil was enriched with many OTUs including, *Saitozyma*, *Trichoderma*, and *Paraglomus* (Supplementary Figure 2C). The functions of the fungal OTUs enriched in W&T, W, and T are described in Supplementary Table 1.

### Co-occurring Network Analysis of Soil Characteristics and Microbial Communities

**FIGURE 8:**
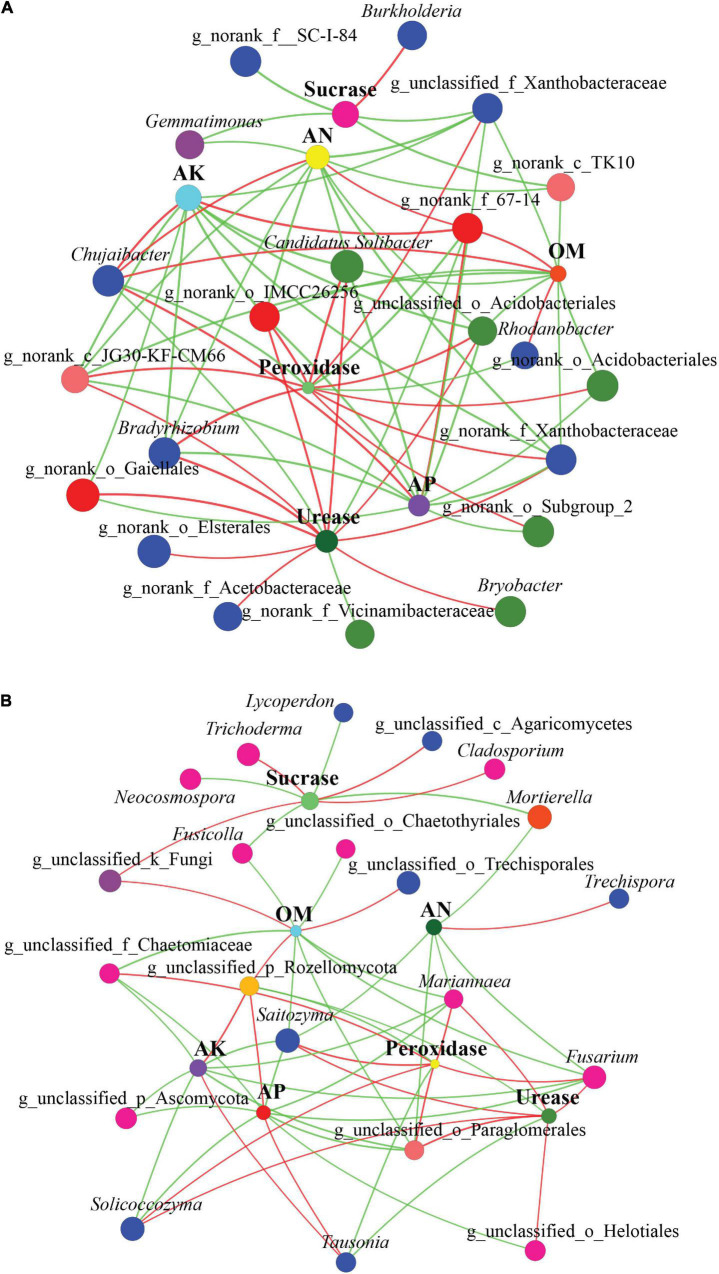
Two-way co-occurring network analysis of soil characteristics, enzyme activity, and bacterial **(A)** and fungal **(B)** community within three forest types. The size of each node is proportional to the relative abundance. The colors of the lines indicate positive (red) and negative (green) correlations.

We constructed two-way co-occurring networks for soil characteristics (soil chemical properties and enzymatic activities) and microbial communities (top 30 genera) (Figures 8A,B). On average, the shortest path length between two nodes consisted of 2.41 edges, with a diameter of 6 edges. We observed 148 degrees of connectivity between soil characteristics and bacterial community across all samples (Figure 8A). In terms of degree centrality scores, the degree of connectivity of soil characteristics was in the order Urease (13) > AP = Peroxidase (12) > AN = OM (11) > AK (10) > Sucrase (5), which indicates the importance of these soil characteristics in the co-occurrence network. In network analysis, a number of bacterial genera showed positive correlations with AP, AN, OM, and AK. Urease and peroxidase activities, however, correlated negatively with many bacterial genera.

In a two-way co-occurrence network analysis of fungal community and soil characteristics, the shortest path between two nodes had an average length of 2.85 edges and a diameter of 6 edges (Figure 8B). Overall, 118 degrees of connectivity were observed between soil characteristics and fungal communities across all samples. Degree centrality scores indicate that soil characteristics are highly interconnected in the order AP = OM (10) > AK (9) > Urease = Peroxidase = Sucrase (8) > AN (6), which indicates the critical role that these soil characteristics play in the co-occurrence network. Moreover, the network analysis showed that some fungal taxa, including *Saitozyma*, *Fusarium*, g_unclassified_o_Paraglomerales, *Mariannaea*, g_unclassified_p_Rozellomycota, *Solicoccozyma*, g_unclassified_f_Chaetomiaceae, and *Tausonia*, had an increased level of connectivity, which suggests that these dominant fungal taxa respond strongly to soil characteristics.

## Discussion

Intercropping has become increasingly popular over the past decade as a way to maintain soil biodiversity, improve nutrient content, and minimize pest and disease problems (Dai et al., 2019). One of the greatest advantages of intercropping is the increase in land-use efficiency and positive interactions between crops, which contribute to their survival, growth, and fitness (Hauggaard-Nielsen and Jensen, 2005). Previous studies have demonstrated that trees within agroforestry systems are capable of improving soil physicochemical properties (Wang et al., 2022). Our results confirmed that soil nutrient contents (AN, AP, AK, and OM) were significantly higher in walnut–tea intercropped forests than in monoculture walnut and tea forests, indicating that growing two crops simultaneously could improve soil nutrient content. Surprisingly, the amount of AN was significantly lower than that of AK and AP in the intercropped forest, indicating the existence of nitrogen competition. The reduced amount of nitrogen contents may be linked to the walnut fruit development, which requires a large amount of nitrogen for the protein synthesis of nuts. On the other hand, a notable increase in AK and AP concentrations was observed in walnut–tea intercropped forests. This might be due to the increased abundance of associated microbial taxa involved in soil nutrient cycling, including rhizobia and phosphate- and potassium-solubilizing bacteria. Another reason for this might be the release of nutrients by agroforestry trees to meet crop demands (Palm, 1995). Potassium can promote nitrogen and phosphorus uptake, leading to lavish vegetative growth, phosphorus translocation, and stimulating photosynthesis that is beneficial to crop growth and biomass production, thereby leading to an increase in yield (Ahmed et al., 2020).

Soil enzyme activity is an important indicator of how the soils cycle nutrients and decompose OM (Nannipieri et al., 2012; Hussain et al., 2021). Soil physicochemical properties and soil microorganisms are known to affect soil enzyme activity (Gu et al., 2009). In this study, we found that intercropped forests had the highest sucrase enzyme activity than monoculture tea and walnut forests. In contrast, peroxidase enzymes activity in the intercropped forest was significantly lower than in monoculture tea and walnut forests. Soil urease activity was significantly lower in intercropped and walnut forests than in monoculture tea forests. Sucrase hydrolyzes sucrose and can reflect soil organic carbon conversion ability, while urease hydrolyzes urea can affect soil nitrogen metabolism (Cantarella et al., 2018). Peroxidase activity decreases with increasing inorganic N availability (Cantarella et al., 2018), which is in alignment with our results. Saccharose catabolism provides a carbon source for microbes, thereby promoting their growth (Ruan, 2014). It appears that intercropped forests can provide sufficient energy for soil microorganisms since they play a key role in the accumulation, decomposition, and transformation of soil organic carbon.

Another focus of our investigation was the comparison of soil microbial communities in walnut–tea intercropping versus monoculture systems. Microorganisms from distinct phylogenetic lineages differ in their response to environmental changes (Hussain et al., 2016; Tian et al., 2018; Zhang W. W. et al., 2019; Zhang et al., 2021); thus, intercropping practices may influence soil microbiota compositions (Bainard et al., 2012a). We observed a profound shift in soil microbial communities when walnut was intercropped with tea. Walnut–tea intercropping caused changes in soil physicochemical properties and enzyme activities, forcing a specific subset of functional bacteria and fungi to enrich in soil, as evidenced by the increase in bacterial and fungal diversity in the intercropping soil relative to tea plantation. Plant species diversity, soil chemistry, and litter quality affect microbial communities (Guo et al., 2018). Studies have demonstrated that microbial diversity increases with vegetation aboveground and with afforestation (Xiao et al., 2016; Liu D. et al., 2018; Liu J. L. et al., 2018; Gao et al., 2019). We speculate that with the extension of natural forest, shading intensifies, resulting in the decrease of canopy temperature and root exudates (Abbasi et al., 2020). Therefore, reasonable perennial non-wood production forests intercrop with crops (walnut/tea) will not only increase soil nutrient contents and enzyme activity but also increase microbial diversity, which will be more favorable for plant growth.

Although intercropped and monoculture walnut forests did not differ in α-diversity, the PCoA of soil bacterial and fungal communities revealed a clear separation between the two forest types, indicating that the microbiota composition differed between monoculture walnut forest and intercropped forest. In this study, Proteobacteria, Actinobacteria, Acidobacteria, Chloroflexi, Bacteroidetes, Firmicutes, and Planctomycetes were the dominant bacterial phyla in the different forest types, which roughly correspond to the results of previous studies investigating agricultural soils (Bai et al., 2020). The walnut–tea intercropped forest had a significantly higher abundance of phyla Proteobacteria, Bacteroidetes, and Firmicutes than the tea and walnut forest soil samples. In contrast, Acidobacteria and Chloroflexi were much less abundant in the intercropping forest than in the tea and walnut forest soil samples. Previous reports indicate that many members of the phyla of Proteobacteria and Bacteroidetes were closely associated with the C and N cycle (Leff et al., 2015; Pardon et al., 2017). As a result, the intercropping forest might increase soil C and N accumulation and nutrient utilization efficiency by promoting bacterial growth that is closely associated with N fixation or other C–N processes. In addition, we observed a high abundance of Firmicutes in the walnut–tea intercropping soil possibly due to their ability to produce endospore as a result of soil microclimate changes (Gomez-Montano et al., 2013). Firmicutes express transcripts for glycosylase hydrolases involved in cellulose and chitin breakdown (Wegner and Liesack, 2016). A striking pattern was that the abundance of Nitrospirae in the monoculture walnut forest was more than in the monoculture tea forest, which are known to be involved in ammonia-oxidizing and nitrite-oxidizing processes (Beeckman et al., 2018). According to previous studies, the decline in the RA of Nitrospirae is due to tea trees that prefer NH_4_^+^, which reduces the ammonia-oxidizing and nitrite-oxidizing processes (Ruan et al., 2016). Gemmatimonadetes are facultative bacteria (Takaichi et al., 2010) and have been significantly correlated with soil OM and N content (Chen et al., 2016). Chloroflexi were found to be abundant because they produce energy by solar radiation and 3-hydroxypropionate bi-cycle (Berg, 2011; Klatt et al., 2013). Therefore, Chloroflexi are more abundant in individual forests due to open canopy that receives more sunlight than intercropping forest (Tripathi et al., 2016). Chloroflexi and Acidobacteria have previously been shown to be slow-growing bacteria (Davis et al., 2011) that prefer oligotrophic environments (Fierer et al., 2012). Because of the oligotrophic environment in the monocropping forest, the RA of Chloroflexi and Acidobacteria was higher than in intercropped forests (Zhu et al., 2019; Xu et al., 2020). Importantly, the significantly higher abundance of Proteobacteria in the intercropping forest compared to monocropping forests is indicative of eutrophic soils (Smit et al., 2001), pointing out that soil nutrient status was improved after intercropping.

In walnut–tea intercropping forest, we detected several enriched bacterial OTUs associated with phosphorus and potassium solubilization. They belonged to the bacterial genera *Pseudomonas*, *Rhizobium*, *Arthrobacter*, *Sphingomonas*, *Bacillus*, and *Burkholderia*, which are known for their functions such as plant growth promotion, phosphorus-solubilizing, nitrogen-fixing, degradation and biotransformation of organic compounds, and growth stimulation (Rodrıìguez and Fraga, 1999; Chen et al., 2006; Sharma et al., 2013; Panhwar et al., 2014; Hu et al., 2020; Morya et al., 2020; Paulitsch et al., 2020; Sadauskas et al., 2020; Tapia-García et al., 2020; Yang et al., 2020). Similarly, *Bacillus* and *Burkholderia* have demonstrated the ability to solubilize potassium (Basak and Biswas, 2009; Zhang and Kong, 2014). Moreover, we found a significant enrichment of *Chujaibacter*, *Acidothermus*, *Acidipila*, *Micropepsaceae*, *Bryobacter*, and *Pseudolabrys* in the intercropped forest. *Chujaibacter* is known to be involved in nitrogen cycling reaction–nitrification (Semenov et al., 2020), and its abundances were positively correlated with soil OM, nitrogen, and phosphorus content in the present study (Figure 8). *Acidothermus* is found to be arbuscular mycorrhizal fungi suppressive taxa (Svenningsen et al., 2018), and *Bryobacter* is plant growth-promoting rhizobacteria (Liu et al., 2019). *Pseudolabrys* is highlighted to be able to improve salinity stress (Lee et al., 2021). The intercropping forest also showed significant enrichment of *Rhodanobacter*. Members of *Rhodanobacter* are known as denitrifying bacteria that are used for bioremediation (Prakash et al., 2012).

In the fungal community, Ascomycota and Mortierellomycota were significantly more abundant in monoculture walnut forests than in monoculture tea forests. However, Glomeromycota were more abundant in tea soils than walnut soils and walnut–tea intercropping forests. The roots of tea trees form a symbiotic relationship with arbuscular mycorrhizal fungi in soils containing low levels of inorganic phosphorus (Öpik et al., 2010; Wu et al., 2019). As confirmed in this study, monoculture tea forest soils had a significantly lower amount of AP than in monoculture walnut and intercropping forest. Further research is needed to understand the reasons for the decrease in arbuscular mycorrhizal fungi in walnut forest land or intercropping forest land. Moreover, we found that soils in the intercropping forest contained a significantly higher abundance of *Trechispora*, *Oidiodendron*, *Penicillium*, *Talaromyces*, *Trichoderma*, and *Pochonia* than those in monoculture tea and walnut forests. The higher abundance of *Trechispora* in the soil has been positively correlated with the increased uptake of nitrogen and carbon sources by hosts (Vanegas-León et al., 2019) and litter decomposition (Midgley et al., 2015). Members of the genus *Trichoderma* are known for their ability to reduce the severity of plant diseases by inhibiting pathogenic organisms in the soil through their highly potent antagonistic and mycoparasitic activity (Schuster and Schmoll, 2010; Hermosa et al., 2012; Ghazanfar et al., 2018; Chen et al., 2021). In prior studies, *Oidiodendron* has been demonstrated to improve phosphorus and nitrogen uptake (Vohnfk and Albrechtová, 2005). *Penicillium*, *Talaromyces*, and *Pochonia* species are also shown to inhibit plant pathogens (Elsharkawy et al., 2012; Hamid et al., 2017; Mwaheb et al., 2017; Topalovic et al., 2020; Abdul et al., 2021).

Based on the above discussion, it becomes clear that intercropping walnut and tea positively affects soil nutrient contents, enzyme activity, and the composition of bacterial and fungal microbiota in the soil microbiome and that changes in microbial diversity and RA play an important role in nutrient cycling, which benefits both walnut and tea trees.

## Conclusion

The results of our study suggest that intercropping practices in orchards had a significant impact on improving the soil’s nutritional conditions by increasing soil nitrogen, phosphorus, potassium, and OM. The intercropping practice also induced great changes in soil bacterial and fungal communities. An analysis of microbial and fungal communities confirmed that walnut–tea intercropped had a significant impact on the bacterial and fungal community diversity and composition as compared to the monoculture walnut and tea forests. In general, the intercropping practice significantly optimized bacterial and fungal community structure and harbor relatively enriched beneficial bacterial and fungal taxa that were mainly related to nutrient cycling and disease protection. Nevertheless, the functional potential of soil microbial communities using shotgun metagenomics and metatranscriptomics is needed to further study.

## Data Availability Statement

The datasets presented in this study can be found in online repositories. The names of the repository/repositories and accession number(s) can be found in the article/Supplementary Material.

## Author Contributions

Y-CB and Y-JX conceived the idea. Y-CB, C-YX, B-XL, QW, Q-ZW, and Y-NF collected and prepared the samples for soil, enzyme, and microbiome sequencing analysis. Y-CB, MR, J-YH, AI, and MH performed the bioinformatics analysis and prepared the figures. Y-CB, MH, and Y-JX wrote the manuscript. All authors listed have made a substantial, direct, and intellectual contribution to the work, and approved it for publication.

## Conflict of Interest

J-YH is employed by Shenyang Sinochem Agrochemicals R&D Co., Ltd. The remaining authors declare that the research was conducted in the absence of any commercial or financial relationships that could be construed as a potential conflict of interest.

## Publisher’s Note

All claims expressed in this article are solely those of the authors and do not necessarily represent those of their affiliated organizations, or those of the publisher, the editors and the reviewers. Any product that may be evaluated in this article, or claim that may be made by its manufacturer, is not guaranteed or endorsed by the publisher.

## Supplementary Material

Supplementary Figure 1 Heat map showing relative abundances of the significantly enriched bacterial OTUs found in the **(A)** walnut-tea intercropping forest (W&T), **(B)** monoculture walnut forest (W), and **(C)** monoculture tea forest (T).

Supplementary Figure 2 Heat map showing relative abundances of the significantly enriched fungal OTUs found in the **(A)** walnut-tea intercropping forest (W&T), **(B)** monoculture walnut forest (W), and **(C)** monoculture tea forest (T).
