# Supplementary figures and images for: Intercropping Walnut and Tea: Effects on Soil Nutrients, Enzyme Activity, and Microbial Communities
Source: Front Microbiol. 2022 Mar 18;13:852342. doi: 10.3389/fmicb.2022.852342 (PMC8971985; doi:10.3389/fmicb.2022.852342)

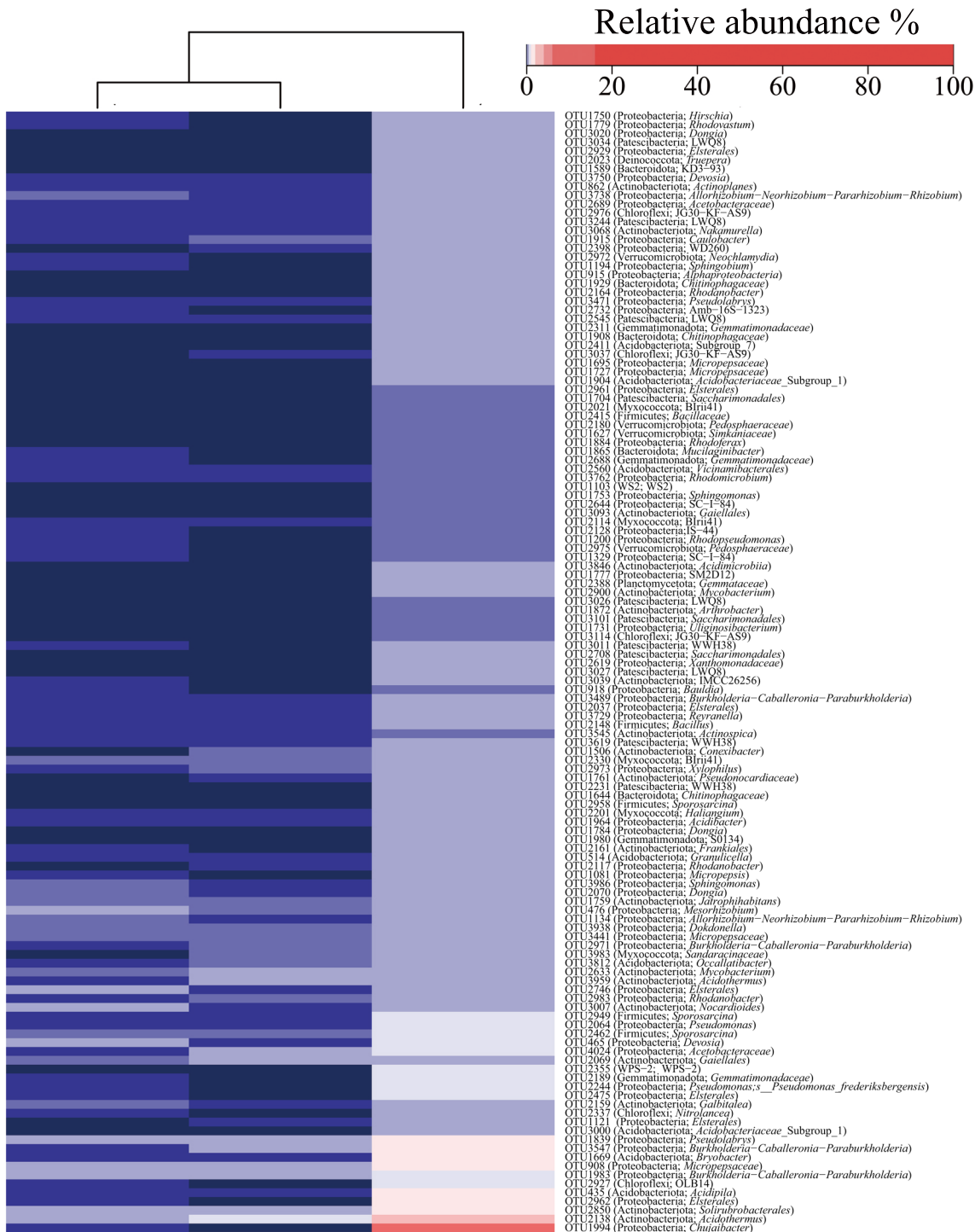

W

T

W&amp;T

Supplement: Supplementary Figure 1 — Heat map showing relative abundances of the significantly enriched bacterial OTUs found in the (A) walnut-tea intercropping forest (W&T), (B) monoculture walnut forest (W), and (C) monoculture tea forest (T). [file Data_Sheet_1.PDF]

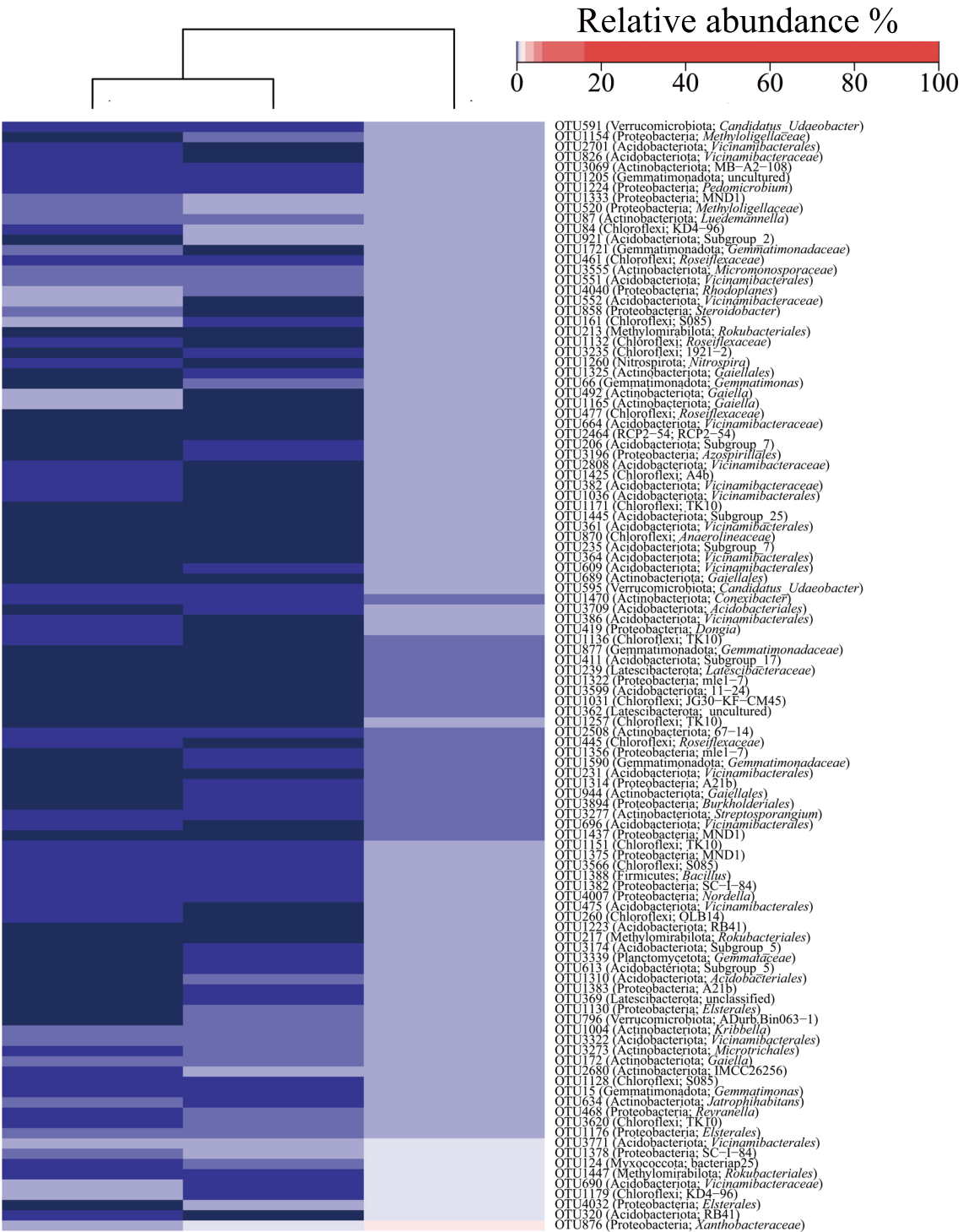

W&T

T

W

Supplement: Supplementary Figure 2 — Heat map showing relative abundances of the significantly enriched fungal OTUs found in the (A) walnut-tea intercropping forest (W&T), (B) monoculture walnut forest (W), and (C) monoculture tea forest (T). [file Data_Sheet_2.PDF]

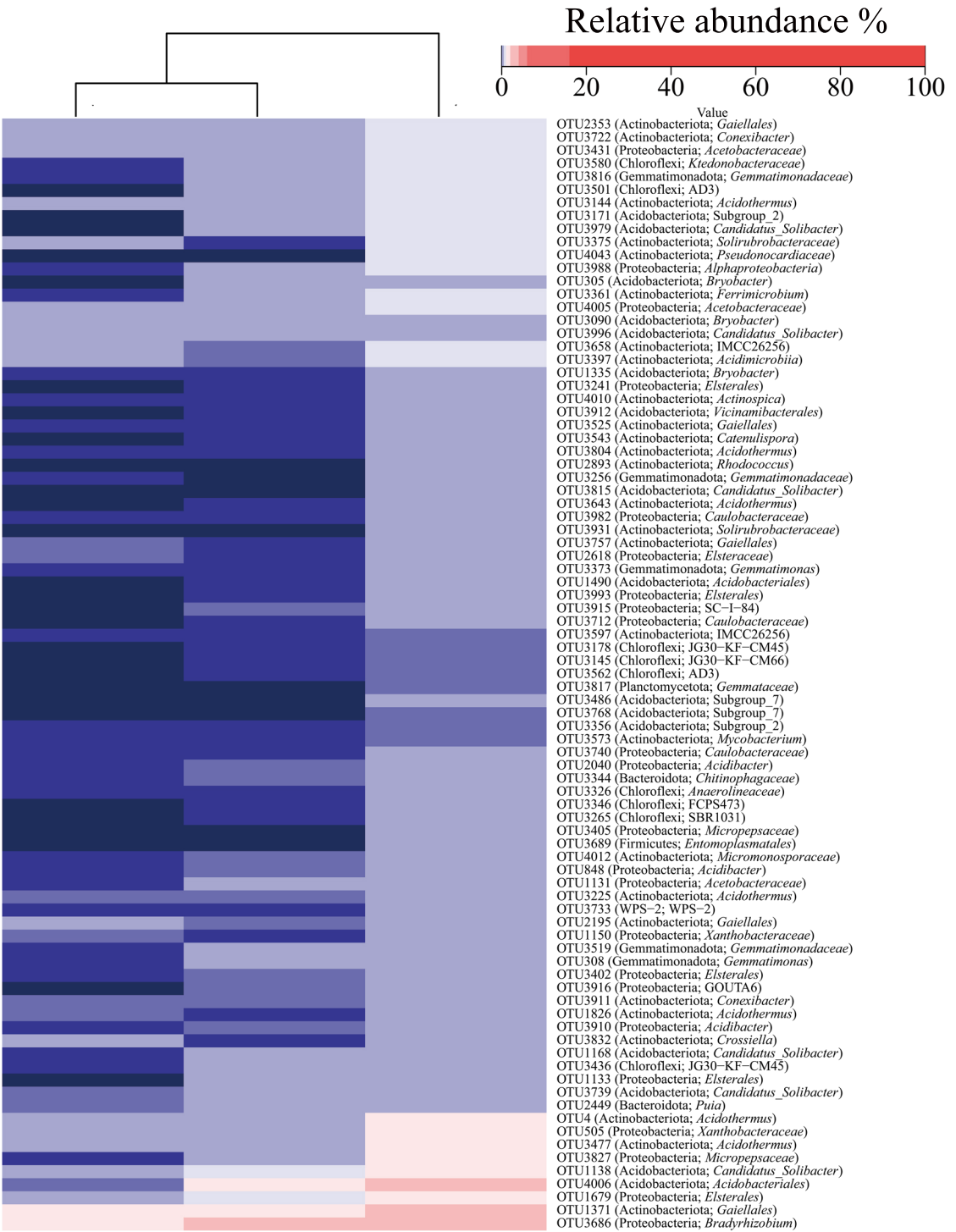

W&T

W

T

Supplement: Supplementary file 3 [file Data_Sheet_3.PDF]

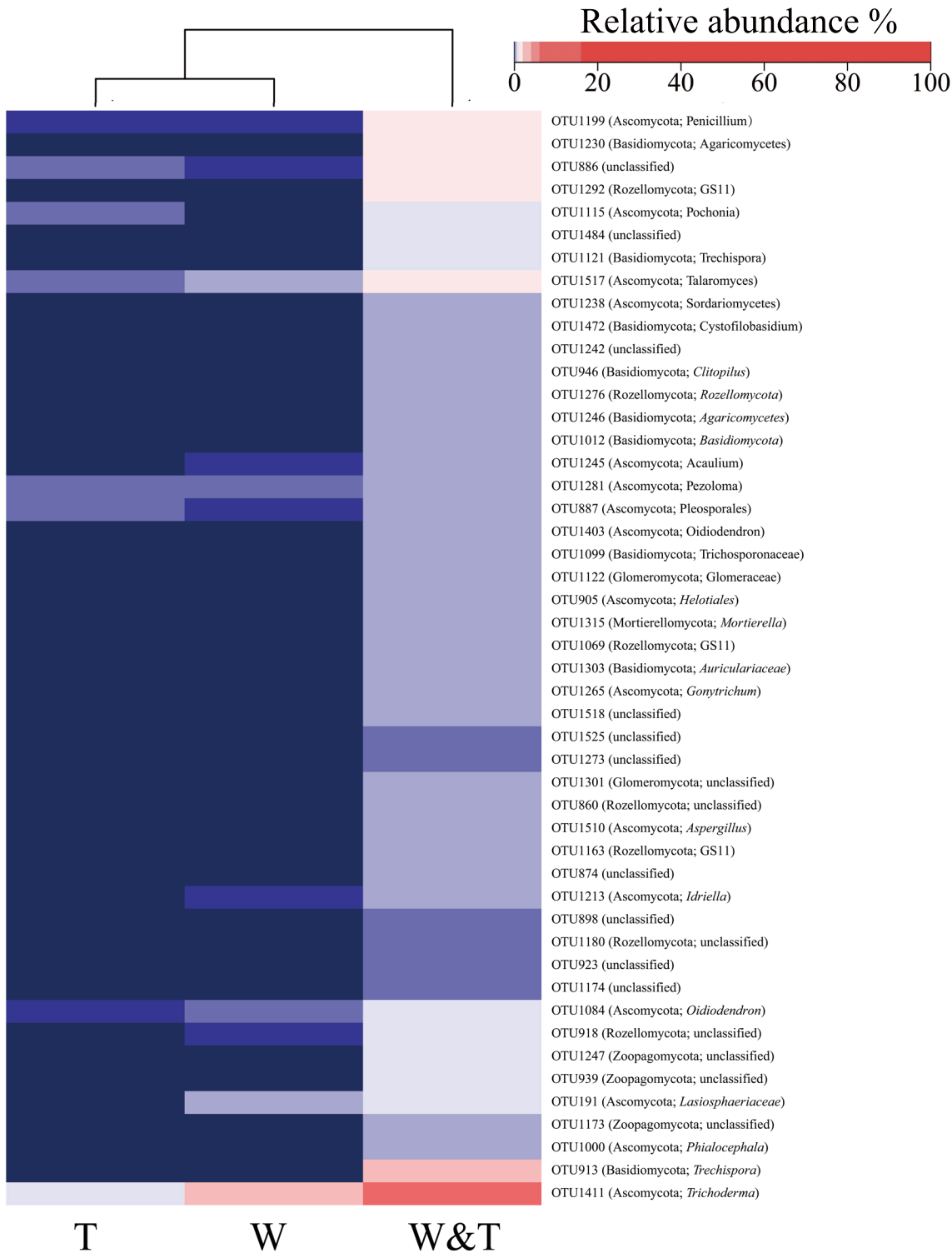

Supplement: Supplementary file 4 [file Data_Sheet_4.PDF]

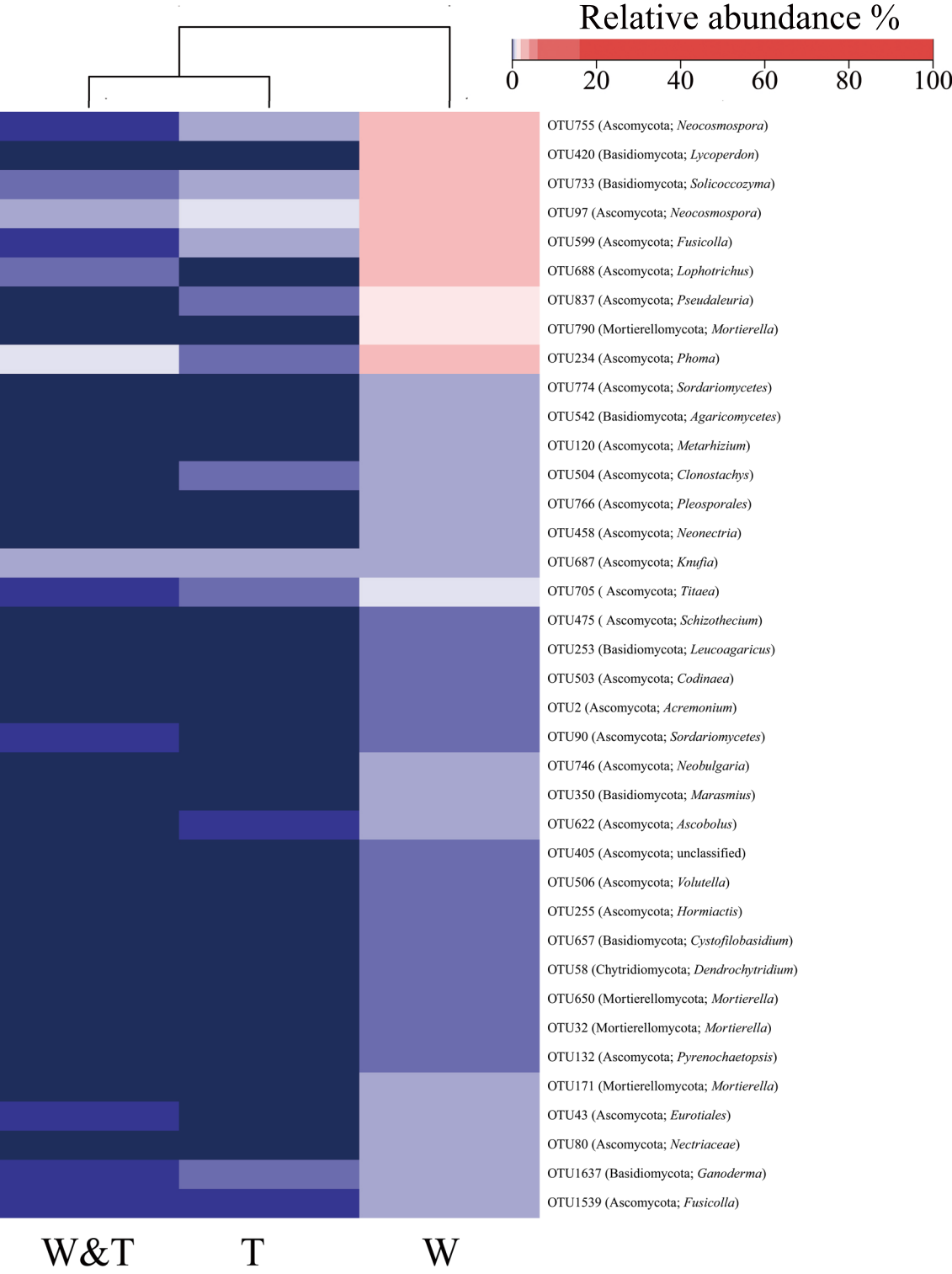

Supplement: Supplementary file 5 [file Data_Sheet_5.PDF]

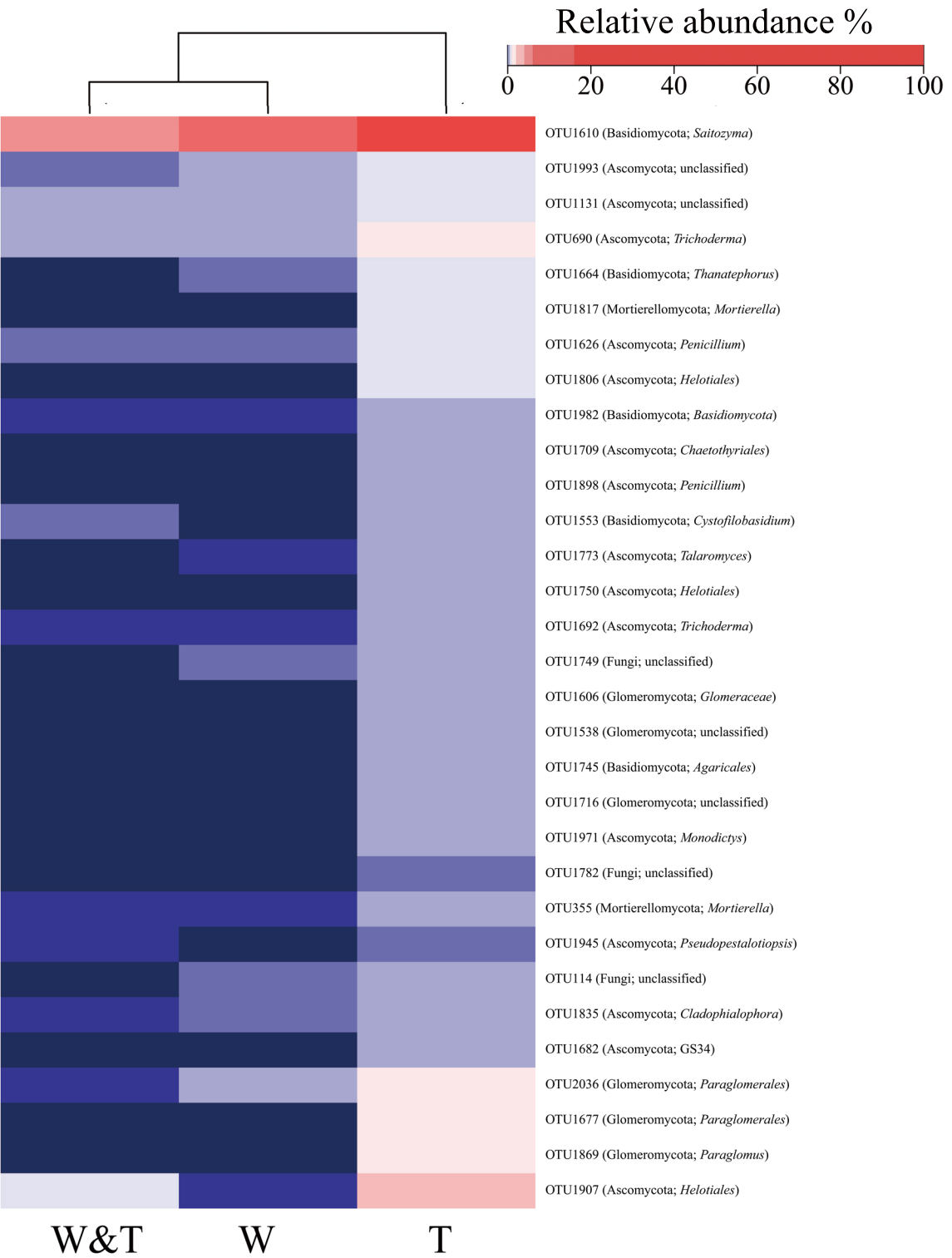

Supplement: Supplementary file 6 [file Data_Sheet_6.PDF]
