# Supplementary material for: Intercropping Walnut and Tea: Effects on Soil Nutrients, Enzyme Activity, and Microbial Communities
Source: Front Microbiol. 2022 Mar 18;13:852342. doi: 10.3389/fmicb.2022.852342 (PMC8971985; doi:10.3389/fmicb.2022.852342)
Supplement: Supplementary file 7 [file Table_1.DOCX]

Table S1: Putative function of some of the dominant bacterial and fungal enriched OTUs in the soil microbiome across three forest types.

| **W&T Enriched bacterial OTUs** | | | |
| --- | --- | --- | --- |
| OTU Number | Genera | Function | Reference |
| OTU465 | *Devosia* | Nitrogen-fixing bacteria | Sprent *et al*., 2017 |
| OTU1669 | *Bryobacter* | Plant growth promoting rhizobacteria; Biogeochemical carbon cycle | Liu *et al*., 2019; Dedysh *et al*., 2016 |
| OTU1839 | *Pseudolabrys* | Improving salinity stress | Lee *et al*., 2021 |
| OTU1983; OTU3547 | *Burkholderia-Caballeronia-Paraburkholderia* | Plant growth promoting rhizobacteria; Phosphorus-solubilizing, nitrogen-fixing, degradation and biotransformation of organic compounds, and growth stimulating | Morya *et al*., 2020; Paulitsch *et al*., 2020; Sadauskas *et al*., 2020; Tapia-Garcia *et al*., 2020b; Yang *et al*., 2020; Ravi *et al*., 2021 |
| OTU1994 | *Chujaibacter* | Nitrogen cycling reaction- nitrification | Semenov *et al*., 2020 |
| OUT 2138 | *Acidothermus* | AMF-suppressive | Svenningsen *et al*., 2018 |
| OTU2462 | *Sporosarcina* | Produced indole acetic acid and siderophore; solubilize phosphorous and fix atmospheric nitrogen | Janarthine and Eganathan, 2012 |
| OTU2064 | *Pseudomonas* | Plant growth promoting rhizobacteria; Nutrient availability | Akinola *et al*., 2021; Windisch *et al*., 2021 |
| OTU2850 | *Solirubrobacterales* | Carbon cycling reaction | Liu *et al*., 2020 |
|  |  |  |  |
| **W Enriched bacterial OTUs** | | | |
| OTU876 | *Xanthobacteraceae* | Nitrogen cycling | Zhu *et al*., 2018; Jiang *et al*., 2020 |
| OTU1138; OTU3979 | *Candidatus_solibacter* | Nutrient cycling | Bai *et al*., 2020 |
| OTU3686 | *Bradyrhizobium* | Nitrogen-fixing | Hayat *et al*., 2010 |
|  |  |  |  |
| **T Enriched bacterial OTUs** | | | |
| OTU1371; OTU2353 | *Gaiellales* | Decomposed organic matter; Preferences low pH conditions | Lin *et al*., 2019; Svenningsen *et al*., 2017 |
| OTU3144 | *Candidatus_solibacter* | Nutrient cycling | Bai *et al*., 2020 |
| OTU3722 | *Conexibacter* | Nutrient availability | Akinola *et al*., 2021 |
| OTU3361 | *Ferrimicrobium* | Preference low pH conditions | Johnson *et al*., 2009 |
| OTU3686 | *Bradyrhizobium* | Nitrogen-fixing | Barelli *et al*., 2020 |
|  |  |  |  |
| **W&T Enriched fungal OTUs** | | | |
| OTU913 | *Trechispora* | Litter decomposition | Midgley *et al*., 2015 |
| OTU1084 | *Oidiodendron* | Improving phosphorus and nitrogen uptake | Vohnik *et al*., 2005 |
| OTU1199 | *Penicillium* | Improving plant growth or acting as biocontrol agents against plant pathogens | Larena *et al*., 2003; Khan *et al*., 2011; Elsharkawy *et al*., 2012 |
| OTU1292 | *Talaromyces* | Biocontrol | Rahman *et al*., 2021 |
| OTU1411 | *Trichoderma* | Biocontrol, biofertilization, and phytostimulation | Kashyap *et al*., 2017; Kotasthane *et al*. 2015; Hermosa *et al*., 2012 |
|  |  |  |  |
| **W Enriched fungal OTUs** | | | |
| OTU97; OTU755 | Neocosmospora (*Fusarium*) | Pathogens | Sandoval-Denis *et al*., 2018 |
| OTU234 | *Phoma* | Biocontrol; Improving salinity stress | Hassan *et al*. 2019; Waqas *et al*. 2012 |
| OTU420 | *Lycoperdon* | pathogens | Inguagiato *et al*., 2015 |
| OTU599 | *Fusicolla* | Plant growth promoting rhizobacteria | Lay *et al*., 2018 |
| OTU790 | *Mortierella* | Plant growth promoting rhizobacteria | Windisch *et al*., 2021; Kamzolova *et al*., 2014; Tamayo-Velez and Osorio, 2017; Li *et al*., 2018 |
|  |  |  |  |
| **T Enriched fungal OTUs** | | | |
| OTU355 | *Mortierella* | Plant growth promoting rhizobacteria | Windisch *et al*., 2021; Kamzolova *et al*., 2014; Tamayo-Velez and Osorio, 2017; Li *et al*., 2018 |
| OTU1664 | *Thanatephorus* | Pathogens | Nassimi and Taheri 2017, |
| OTU1806 | Helotiales (*Botrytis*) | Pathogens | Vrålstad *et al*., 2002 |

References:

Abdul Rahman N S N, Abdul Hamid N W, Nadarajah K. Effects of abiotic stress on soil microbiome[J]. International Journal of Molecular Sciences, 2021, 22(16): 9036.

Akinola S A, Ayangbenro A S, Babalola O O. Metagenomic Insight into the Community Structure of Maize-Rhizosphere Bacteria as Predicted by Different Environmental Factors and Their Functioning within Plant Proximity[J]. Microorganisms, 2021, 9(7): 1419.

Bai Y C, Chang Y Y, Hussain M, *et al*. Soil chemical and microbiological properties are changed by long-term chemical fertilizers that limit ecosystem functioning[J]. Microorganisms, 2020, 8(5): 694.

Barelli L, Waller A S, Behie S W, *et al*. Plant microbiome analysis after Metarhizium amendment reveals increases in abundance of plant growth-promoting organisms and maintenance of disease-suppressive soil[J]. PloS one, 2020, 15(4): e0231150.

Dedysh S N, Kulichevskaya I S, Huber K J, *et al*. Defining the taxonomic status of described subdivision 3 Acidobacteria: proposal of Bryobacteraceae fam. nov[J]. International journal of systematic and evolutionary microbiology, 2017, 67(2): 498-501.

Vrålstad T, Myhre E, Schumacher T. Molecular diversity and phylogenetic affinities of symbiotic root‐associated ascomycetes of the Helotiales in burnt and metal polluted habitats[J]. New Phytologist, 2002, 155(1): 131-148.

Elsharkawy M M, Shimizu M, Takahashi H, *et al*. Induction of systemic resistance against Cucumber mosaic virus by Penicillium simplicissimum GP17‐2 in Arabidopsis and tobacco[J]. Plant Pathology, 2012, 61(5): 964-976.

Hassan S E D, Fouda A, Radwan A A, *et al*. Endophytic actinomycetes Streptomyces spp mediated biosynthesis of copper oxide nanoparticles as a promising tool for biotechnological applications[J]. JBIC Journal of Biological Inorganic Chemistry, 2019, 24(3): 377-393.

Hayat R, Ali S, Amara U, *et al*. Soil beneficial bacteria and their role in plant growth promotion: a review[J]. Annals of microbiology, 2010, 60(4): 579-598.

Hermosa R, Viterbo A, Chet I, *et al*. Plant-beneficial effects of Trichoderma and of its genes[J]. Microbiology, 2012, 158(1): 17-25.

Inguagiato J C, Martin S B. Diseases of cool-and warm-season putting greens[J]. Green Sect. Rec, 2015, 53(9): 1-19.

Jain P, Pundir R K. Potential role of endophytes in sustainable agriculture-recent developments and future prospects[J]. Endophytes: biology and biotechnology, 2017: 145-169.

Jiang H, Shao H, Xiang Q, *et al*. Continuous Cropping and Natural Fallow Practices Affect Tobacco Fitness and Soil Microbiomes[J]. Authorea Preprints, 2020.

Johnson D B, Bacelar-Nicolau P, Okibe N, *et al*. Ferrimicrobium acidiphilum gen. nov., sp. nov. and Ferrithrix thermotolerans gen. nov., sp. nov.: heterotrophic, iron-oxidizing, extremely acidophilic actinobacteria[J]. International journal of systematic and evolutionary microbiology, 2009, 59(5): 1082-1089.

Kamzolova S V, Vinokurova N G, Dedyukhina E G, *et al*. The peculiarities of succinic acid production from rapeseed oil by Yarrowia lipolytica yeast[J]. Applied microbiology and biotechnology, 2014, 98(9): 4149-4157.

Kashyap P L, Rai P, Srivastava A K, *et al*. Trichoderma for climate resilient agriculture[J]. World Journal of Microbiology and Biotechnology, 2017, 33(8): 1-18.

Khan A L, Hamayun M, Kim Y H, *et al*. Ameliorative symbiosis of endophyte (Penicillium funiculosum LHL06) under salt stress elevated plant growth of Glycine max L[J]. Plant Physiology and Biochemistry, 2011, 49(8): 852-861.

Kotasthane A, Agrawal T, Kushwah R, *et al*. In-vitro antagonism of Trichoderma spp. against Sclerotium rolfsii and Rhizoctonia solani and their response towards growth of cucumber, bottle gourd and bitter gourd[J]. European Journal of Plant Pathology, 2015, 141(3): 523-543.

Larena I, Sabuquillo P, Melgarejo P, *et al*. Biocontrol of Fusarium and Verticillium wilt of tomato by Penicillium oxalicum under greenhouse and field conditions[J]. Journal of Phytopathology, 2003, 151(9): 507-512.

Lay C Y, Bell T H, Hamel C, *et al*. Canola root–associated microbiomes in the Canadian Prairies[J]. Frontiers in microbiology, 2018, 9: 1188.

Lee S A, Kim H S, Sang M K, *et al*. Effect of Bacillus mesonae H20-5 Treatment on Rhizospheric Bacterial Community of Tomato Plants under Salinity Stress[J]. The Plant Pathology Journal, 2021, 37(6): 662.

Li F, Chen L, Redmile‐Gordon M, *et al*. Mortierella elongata's roles in organic agriculture and crop growth promotion in a mineral soil[J]. Land Degradation & Development, 2018, 29(6): 1642-1651.

Liu K, Cai M, Hu C, *et al*. Selenium (Se) reduces Sclerotinia stem rot disease incidence of oilseed rape by increasing plant Se concentration and shifting soil microbial community and functional profiles[J]. Environmental Pollution, 2019, 254: 113051.

Liu Y R, Delgado-Baquerizo M, Yang Z, *et al*. Microbial taxonomic and functional attributes consistently predict soil CO2 emissions across contrasting croplands[J]. Science of The Total Environment, 2020, 702: 134885.

Lin Y, Ye G, Kuzyakov Y, *et al*. Long-term manure application increases soil organic matter and aggregation, and alters microbial community structure and keystone taxa[J]. Soil Biology and Biochemistry, 2019, 134: 187-196.

Midgley M G, Brzostek E, Phillips R P. Decay rates of leaf litters from arbuscular mycorrhizal trees are more sensitive to soil effects than litters from ectomycorrhizal trees[J]. Journal of Ecology, 2015, 103(6): 1454-1463.

Morya R, Salvachúa D, Thakur I S. Burkholderia: an untapped but promising bacterial genus for the conversion of aromatic compounds[J]. Trends in Biotechnology, 2020.

Nassimi Z, Taheri P. Endophytic fungus Piriformospora indica induced systemic resistance against rice sheath blight via affecting hydrogen peroxide and antioxidants[J]. Biocontrol Science and Technology, 2017, 27(2): 252-267.

Paulitsch F, Dall’Agnol R F, Delamuta J R M, *et al*. Paraburkholderia atlantica sp. nov. and Paraburkholderia franconis sp. nov., two new nitrogen-fixing nodulating species isolated from Atlantic forest soils in Brazil[J]. Archives of microbiology, 2020, 202(6): 1369-1380.

Ravi A, Theresa M, Nandayipurath V V T, *et al*. Plant beneficial features and application of Paraburkholderia sp. NhPBG1 isolated from pitcher of Nepenthes hamblack[J]. Probiotics and Antimicrobial Proteins, 2021, 13(1): 32-39.

Sadauskas M, Statkevičiūtė R, Vaitekūnas J, *et al*. Bioconversion of biologically active indole derivatives with indole-3-acetic acid-degrading enzymes from Caballeronia glathei DSM50014[J]. Biomolecules, 2020, 10(4): 663.

Sandoval-Denis M, Guarnaccia V, Polizzi G, *et al*. Symptomatic Citrus trees reveal a new pathogenic lineage in Fusarium and two new Neocosmospora species[J]. Persoonia: Molecular Phylogeny and Evolution of Fungi, 2018, 40: 1.

Semenov M V, Krasnov G S, Semenov V M, *et al*. Long-term fertilization rather than plant species shapes rhizosphere and bulk soil prokaryotic communities in agroecosystems[J]. Applied Soil Ecology, 2020, 154: 103641.

Sprent J I, Ardley J, James E K. Biogeography of nodulated legumes and their nitrogen‐fixing symbionts[J]. New Phytologist, 2017, 215(1): 40-56.

Svenningsen N B, Watts-Williams S J, Joner E J, *et al*. Suppression of the activity of arbuscular mycorrhizal fungi by the soil microbiota[J]. The ISME Journal, 2018, 12(5): 1296-1307.

Tamayo-Velez A, Osorio N W. Co-inoculation with an arbuscular mycorrhizal fungus and a phosphate-solubilizing fungus promotes the plant growth and phosphate uptake of avocado plantlets in a nursery[J]. Botany, 2017, 95(5): 539-545.

Tapia-García E Y, Arroyo-Herrera I, Rojas-Rojas F U, *et al*. Paraburkholderia lycopersici sp. nov., a nitrogen-fixing species isolated from rhizoplane of Lycopersicon esculentum Mill. var. Saladette in Mexico[J]. Systematic and Applied Microbiology, 2020, 43(6): 126133.

Vohnfk M, Albrechtová J. The inoculation with Oidiodendron maius and Phialocephala fortinii alters phosphorus and nitrogen uptake, foliar C: N ratio and root biomass distribution in Rhododendron cv. Azurro[J]. Symbiosis, 2005.

Waqas M, Khan A L, Kamran M, *et al*. Endophytic fungi produce gibberellins and indoleacetic acid and promotes host-plant growth during stress[J]. Molecules, 2012, 17(9): 10754-10773.

Windisch S, Sommermann L, Babin D, *et al*. Impact of long-term organic and mineral fertilization on rhizosphere metabolites, root–microbial interactions and plant health of lettuce[J]. Frontiers in microbiology, 2021: 3157.

Yang A, Akhtar S S, Fu Q, *et al*. Burkholderia Phytofirmans PsJN Stimulate Growth and Yield of Quinoa under Salinity Stress[J]. Plants, 2020, 9(6): 672.

Zeilinger S, Gruber S, Bansal R, *et al*. Secondary metabolism in Trichoderma–chemistry meets genomics[J]. Fungal biology reviews, 2016, 30(2): 74-90.

Zhu B K, Fang Y M, Zhu D, *et al*. Exposure to nanoplastics disturbs the gut microbiome in the soil oligochaete Enchytraeus crypticus[J]. Environmental Pollution, 2018, 239: 408-415.
